# Pedestrian Road-Crossing Behaviours: A Protocol for an Explanatory Mixed Methods Study

**DOI:** 10.5539/gjhs.v8n5p27

**Published:** 2015-08-20

**Authors:** Mina Hashemiparast, Ali Montazeri, Saharnaz Nedjat, Reza Negarandeh, Roya Sadeghi, Masoumeh Hosseini, Gholamreza Garmaroudi

**Affiliations:** 1 Department of Health Promotion and Education, School of Public Health, Tehran University of Medical Sciences, Tehran, Iran; 2 Institute for Health Sciences Research, ACECR, Tehran, Iran; 3 Epidemiology and Biostatistics Department, School of Public Health, Knowledge Utilization Research Centre, Tehran University of Medical Sciences, Tehran, Iran; 4 Nursing and Midwifery Care Research Canter, School of Nursing, Tehran University of Medical Sciences, Tehran, Iran; 5 Department of Health Promotion and Education, School of Public Health, Tehran University of Medical Sciences, Tehran, Iran; 6 Department of Health Management and Economics, School of Public Health, Tehran University of Medical Sciences, Tehran, Iran

**Keywords:** explanatory mixed methods, road-crossing behaviours, study pedestrian

## Abstract

**Background:**

Pedestrian crossing is an important traffic safety concern. The aim of this paper is to report the protocol for a sequential explanatory mixed methods study that set out to determine the pedestrians’ traffic behaviors, the associated factors and exploring the perception of young people about the traffic risky behaviors in crossing the road. The ultimate purpose of the study is to design a preventive and cultural based strategy to promote young people’s health.

**Methods:**

This is a sequential explanatory mixed methods design. The study has two sequential phases. During the first phase, a population-based cross-sectional survey of a sample of young people will be conducted using the proportional random multistage cluster sampling method, in Tehran, Iran. Data will be collected by a questionnaire including items on socio-demographic information, items on measuring social conformity tendency, and questions on subjective norms, attitudes, and perceived behavioral control based on the Theory of Planned behavior. In the second phase, a qualitative study will be conducted. A purposeful sampling strategy will be used and participants who can help to explain the quantitative findings will be selected. Data collection in qualitative phase will be predominately by individual in-depth interviews. A qualitative content analysis approach will be undertaken to develop a detailed understanding of the traffic risky behaviors among young pedestrians.

**Conclusion:**

The findings of this explanatory mixed methods study will provide information on traffic risky behaviors in young pedestrians. The findings will be implemented to design a cultural based strategy and intervention programs.

## 1. Introduction

Traffic accidents are a major public health problem worldwide. About 85 percent of road traffic accidents occur in poor and middle-income countries that is equivalent to 90 percent of the annual disability adjusted life years (DALYs) (Leandro, 2012). In low- and middle-income countries, the annual cost of road traffic injuries is between 1 to 3 percent of Gross Domestic Product (GDP) (Kumar et al., 2012). The cost of road traffic injuries in Iran annually is about 2.19 percent of GDP (Rezaei et al., 2013).

Pedestrians are one of the most vulnerable road users and are therefore more at risk than the other users such as cyclists, users of motorized two-or three- wheelers (World Health Organization, 2009). Annually more than 270 thousand pedestrians are killed in accidents and 22 percent of the world’s road fatalities are pedestrians (Hamed, 2001). In Iran also the fatality rate among pedestrians involving in the accidents is more than 30 percent (Rahimi et al., 2012). In addition to the vulnerability of pedestrians, in many cases, pedestrian-vehicle crashes are often the result of poor decisions or risky behaviors exhibited during road crossings’ (Zhou et al., 2009). Ulfarsson et al. investigated fault in pedestrian-motor vehicle crashes in North Carolina and found that the pedestrians’ fault was 59 percent while drivers’ fault was 32 percent (Ulfarsson et al., 2010). Thus, identifying and understanding the factors that are involved in pedestrians’ risky road-crossing behaviors is important. Based on an American report on pedestrian safety there are many factors that predict vehicle- pedestrian collisions (Rosenbloom, 2009). In this regard, multiple studies have examined the effect of age and gender in road-crossing behavior. For instance, studies have shown that women were less likely to intend to cross the road in risky situations and perceived more risk or as one’s age increases his/her intention to cross the road in potentially dangerous situations reduces (Holland et al.,2007; Tom et al., 2011). Personality factors such as risk perception and conformity tendency are other determinants of pedestrians’ intention in risky road crossing (Zhou et al., 2009). Since the variety of variables affecting pedestrians’ intention to cross the road in risky situations, using a theoretical framework for the study of such variables would be crucial. The current study will use the Theory of Planned Behavior (TPB). The TPB suggest that three constructs (attitude, subjective norms and perceived behavioral control) will predict the intention to perform a given behavior (Francis et al., 2004). Previous studies had shown that the TPB was a valid model in predicting various behaviors. For example, it has been applied to address pedestrians’ intentions to violate traffic regulations. The results showed that there was a significant correlation between the model’s components and pedestrians’ behaviors (Moyano, 2002). Although the most important factor in predicting pedestrians’ mortality is their behavioral patterns, research on accidents mainly focused on the road environment and drivers with little attention to pedestrians’ behaviors (Soltani et al., 2010).

In addition, limited quantitative and even no qualitative studies have been conducted in this regard. Thus in order to provide a better understanding of pedestrians’ risky behaviors, it seems that conducting a study focusing on pedestrians behaviors, especially young pedestrians, is of prime importance. However, since the topic needs more in-depth information, we thought conducting a study that provides both quantitative and qualitative data would be a best way to shade more light on the topic. Moreover, no studies have used the Theory of Planned Behavior (TPB) to explain pedestrians’ intention in crossing the road in potentially risky situations in Iran where many pedestrians die each year. This mixed methods study set out to determine traffic risky behaviors in crossing the roads as well as its determinants such as personality and socio-demographic characteristics and the Theory of Planned Behavior constructs. Furthermore, we will explore young people’s perception of traffic risky behaviors. Finally based on the findings, preventive and cultural based strategy for prevention of pedestrian accidents will be proposed.

### 1.1 Aims

The main goals of this study are:

To determine the pedestrians’ traffic risky behaviors in crossing the roads and to determine the association between pedestrians’ traffic risky behaviors and constructs of the TPB;

To determine the association between pedestrians’ traffic risky behaviors and their personality (perceived risk and conformity tendency);

To explore the perception of young people about the traffic risky behaviors in crossing the roads, and;

To offer strategies for modifying traffic risky behaviors of Iranian young people.

## 2. Methods

### 2.1 Design

**Figure 1:**
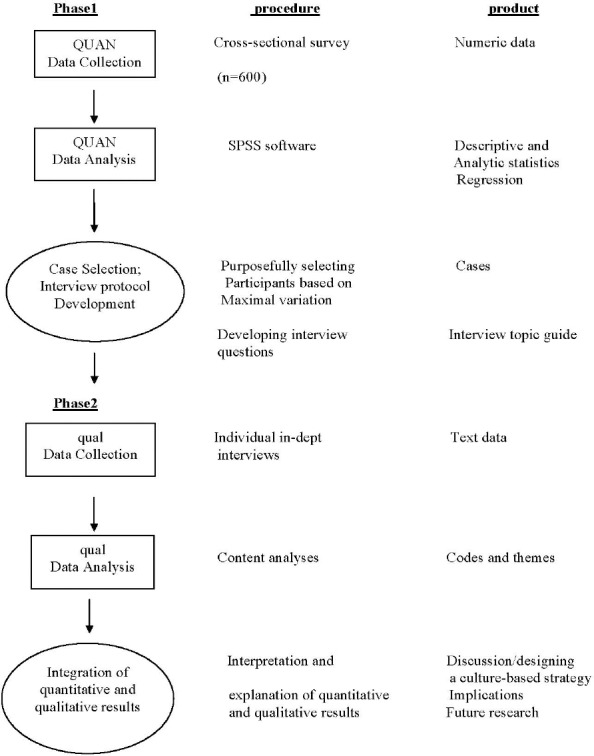
The study framework derived from (Creswell et al., 2011)

This study is designed as sequential explanatory mixed methods that include both quantitative and qualitative methods. The ‘follow-up explanation model’ adopted to explain the quantitative findings by collecting qualitative data (Baheiraei et al., 2011). The explanatory sequential design has two phases. During the first phase, the researcher will collect and analyze quantitative data. The second, qualitative phase will be designed based on the first phase to explain the quantitative findings that need additional explanations such as statistical differences among groups or individuals with unexpected findings. Then, the two phases are connected in the interpretation stage to explain quantitative and qualitative findings (Creswell et al., 2011). The rational for this design is that the quantitative data would provide a general understanding about traffic risky behaviors in young pedestrians and the qualitative data would explore participants’ views about the problem in more depth (Figure 1).

### 2.2 Quantitative Strand

This strand is designed as a population-based cross-sectional study to assess young people traffic risky behaviors in crossing the road in a representative sample of Iranian *18-25* year- old young people.

#### 2.2.1 Sampling

The target population is every 18-25 years old male and female living in Tehran. The required sample size will be calculated based on the mean and standard deviation of the road-crossing risky behaviors in previous studies (mean = 1.33, SD = 0.76) (Yagil, 2000). As such approximately 600 young people will be required for the study. To select a representative sample, a stratified multi-stage area sampling will be applied. Every household within 22 different districts in Tehran would have the same probability of being sampled. For the first stage, units (blocks) will be randomly selected after stratifying by district and size of residence. Then the homes to be sampled within each block will be selected by random routes (one-in-tenth). Finally, the last-stage sampling units (an 18-25 years old male or female) will be selected and entered into the study.

#### 2.2.2 Measures

The data will be collected using the following instruments:

1). A study specific questionnaire containing two scenarios depicting two potentially dangerous road-crossing behaviors asking about people’s intentions to cross the road in these situations. The questionnaire consists of items guided by the Theory of Planned Behavior (TPB) measuring attitude toward the intended behavior, subjective norms, perceived behavioral control and perceived risk.

2). A measure of personality namely The Conformity Tendency Questionnaire (Mehrabian et al., 1995).

This is a questionnaire that contains 10 items and measures social conformity. In fact the questionnaire indicates the extent to which people would follow other people’s behavior when deciding to perform a particular behavior. Questions are rated on a five-point Liker scale giving a total score ranging from 10 to 50.

#### 2.2.3 Data Analyses

Descriptive statistics will be used to explore the quantitative data. Multiple linear regression analysis will be used to assess association between independent variables including TPB constructs traffic risky behaviors as outcome.

### 2.3 Qualitative Strand

#### 2.3.1 Sampling

A purposeful sampling strategy will be used in order to explain quantitative findings.

#### 2.3.2 Data Collection

Individual in-depth interviews using a pre-established interview guide will be held. Focus group discussions will be conduct if required. Before any interviews we will provide recording equipment. The data will be recorded through tape recording and note taking. Interviews will be continued until data saturation and no new themes related to the phenomenon under study emerge.

#### 2.3.3 Data Analyses

We will conduct the content analysis approach. Codes will be generated and grouped based on their similarity into categories to reveal the *18-25* year- old young people perceptions of traffic risky behaviors in crossing the road. Peer and member check will be performed to assess trustworthiness of the data (Krefting, 1991; Ulin et al., 2004). Qualitative data will be managed by using MAXQDA10 software.

### 2.4 Data Integration

According to the sequential explanatory design of the study, we will analyze the quantitative and qualitative components separately because the qualitative phase depends on the quantitative phase (Mbuagbaw et al., 2013). Then, the two phases will be integrated in order to interpret findings.

### 2.5 Ethics

The protocol for this study was approved ethics committee of Tehran University of Medical Sciences. Written informed consent will obtained from all participants before the study commence.

## 3. Discussion

Primary prevention and health promotion could have an important role in accident and injury prevention. Accident and injury prevention is a global concern (Eid et al., 2014).

The main strength of this study is the use of both quantitative and qualitative data that will provide a better understanding of the topic. The combination of quantitative and qualitative methods, allow us to understand the reasons, perceptions and attitude of pedestrians about their risky crossing behaviors in potentially dangerous situations (Hıjar et al., 2003).

It is hoped that this study will provide comprehensive basic information about the young pedestrians’ traffic risky behaviors. In addition, the study will explore behavioral risk related factors to develop effective strategies and interventions. The study results are expected to help policy makers, health promotion department of ministry of health, rode ministry and police in due course.
